# Clinical measurement of sagittal trunk curvatures: photographic angles versus rippstein plurimeter angles in healthy school children

**DOI:** 10.1186/1748-7161-9-S1-O15

**Published:** 2014-12-04

**Authors:** Łukasz Stoliński, Dariusz Czaprowski, Mateusz Kozinoga, Tomasz Kotwicki

**Affiliations:** 1 Rehasport Clinic, Spine Disorders Unit, Department of Pediatric Orthopedics and Traumatology, University of Medical Sciences, Poznań, Poland; 2 Department of Physiotherapy, Józef Rusiecki University College, Olsztyn, Poland; 3 Spine Disorders Unit, Department of Pediatric Orthopedics and Traumatology, University of Medical Sciences, Poznań, Poland

## Background

Digital photography is a simply method to calculate quantitative photographic parameters of the body posture in the frontal and sagittal plane.

## Aim

The aim of the study was to determine the correlation between the measurements of the sagittal trunk curvatures carried out with two diagnostic tools: photography and Rippstein plurimeter.

## Design

This is a reliability study.

## Methods

Sixty-one asymptomatic children (31 girls, 30 boys) aged 7-9 years (mean 7.9 ±0.8) were assessed once by one observer for the sagittal curvatures of the trunk: thoracic kyphosis (TK), lumbar lordosis (LL) and sacral slope (SS) first with digital photography and with Rippstein plurimeter. Statistical analysis was performed using paired Student t-test, Wilcoxon matched-pairs and Pearson correlation coefficient.

## Competing interests

There was no conflict of interest in relation to this study.

## Results

There was no significant difference regarding the measurement of TK performed with photography versus plurimeter (43.3° ±8.8 vs. 43.0° ±8.4, p=0.47). Differences were found for LL (39.8° ±8.2 vs.38.3° ±8.5, p<0.0001) and SS (23.3° ±6.0 vs. 22.7° ±6.4, p=0.024). Significant correlation between measurements performed with photography versus Rippstein plurimeter were observed: TK (r=0.949, p<0.0001), LL (r=0.951, p<0.0001) and SS (r=0.944, p<0.0001).

## Conclusions

Although significant difference for LL and SS were found, the difference between measurements is small, so it seems that photography and Rippstein plurimeter can be used for assessment of sagittal trunk curvatures in the clinical assessment.
